# Lateral Flow Immunoassay Based on Time-Resolved Fluorescence Microspheres for Rapid and Quantitative Screening CA199 in Human Serum

**DOI:** 10.3390/ijms23179991

**Published:** 2022-09-01

**Authors:** Xueshima Jiao, Tao Peng, Zhanwei Liang, Yalin Hu, Bo Meng, Yang Zhao, Jie Xie, Xiaoyun Gong, You Jiang, Xiang Fang, Xiaoping Yu, Xinhua Dai

**Affiliations:** 1 College of Life Sciences, China Jiliang University, Hangzhou 310018, China; 2 Center for Advanced Measurement Science, National Institute of Metrology, Beijing 100029, China

**Keywords:** time-resolved fluorescent microspheres, lateral flow immunoassay, carbohydrate antigen 199, biomarker, cancer

## Abstract

Carbohydrate antigen 199 (CA199) is a serum biomarker which has certain value and significance in the diagnosis, prognosis, treatment, and postoperative monitoring of cancer. In this study, a lateral flow immunoassay based on europium (III) polystyrene time-resolved fluorescence microspheres (TRFM-based LFIA), integrated with a portable fluorescence reader, has been successfully establish for rapid and quantitative analysis of CA199 in human serum. Briefly, time-resolved fluorescence microspheres (TRFMs) were conjugated with antibody I (Ab1) against CA199 as detection probes, and antibody II (Ab2) was coated as capture element, and a “TRFMs-Ab1-CA199-Ab2” sandwich format would form when CA199 was detected by the TRFM-based LFIA. Under the optimal parameters, the detection limit of the TRFM-based LFIA for visible quantitation with the help of an ultraviolet light was 4.125 U/mL, which was four times lower than that of LFIA based on gold nanoparticles. Additionally, the fluorescence ratio is well linearly correlated with the CA199 concentration (0.00–66.0 U/mL) and logarithmic concentration (66.0–264.0 U/mL) for quantitative detection. Serum samples from 10 healthy people and 10 liver cancer patients were tested to confirm the performances of the point-of-care application of the TRFM-based LFIA, 20.0 U/mL of CA199 in human serum was defined as the threshold for distinguishing healthy people from liver cancer patients with an accuracy of about 60%. The establishment of TRFM-based LFIA will provide a sensitive, convenient, and efficient technical support for rapid screening of CA199 in cancer diagnosis and prognosis.

## 1. Introduction

Carbohydrate antigen 199 (CA199) is a polysaccharide containing oligosaccharide sialic acid antigen and a cancer-associated glycoprotein antigen, which was first isolated from colon and pancreatic cancer by Koprowski and his colleagues in 1979 [1]. The level of CA199 in serum can be significantly increased because of the arising of epithelial malignancy caused by differentiation of endodermal cells [2]. CA199 in serum has been used as an indicator of aberrant glycosylation [3] and a biomarker, predictor, and promoter for the diagnosis, prognosis, and monitoring of pancreatic cancer. Over the past few years, CA199 has been found to also exhibit certain diagnostic values for gastric cancer, pancreatic cancer [4], esophageal cancer [5], liver cancer [6], lung cancer, and ovarian cancer [7] with high sensitivity. Therefore, the sensitive and rapid detection of CA199 is seen to be particularly important for patients in early clinical diagnosis, preoperative staging, assessment of resectability, and evaluation of the recovery. Up to date, a variety of analytical methods, including enzyme-linked immunosorbent assay (ELISA) [8], chemiluminescence immunoassay [9], radioimmunoassay [10], electrochemical immunoassay [11], and lateral flow immunoassay [12], have been developed and applied to detect CA199. Among them, ELISA has been considered the gold standard for detecting CA199 [13], but it suffers from having a time-consuming and tedious operation. In resource-limited or emergency situations, rapid and sensitive detection of CA199 without professional equipment or technical personnel is urgent and necessary for the point-of-care diagnosis of some related diseases.

Lateral flow immunoassay (LFIA), a point-of-care testing (POCT) technology that appeared in the 1990s, is based on the specific reactions between antigens and antibodies. It has been widely used because of its advantages of simplicity, rapidity, convenience, low cost, and high efficacy. The mature LFIA has become a commonly used POCT technique in clinical diagnosis of various diseases, such as early-stage cancer [14], sexually transmitted diseases [15], AIDS [16], COVID-19 [17], and so forth. Our group have successfully developed LFIAs to rapidly detect SARS-CoV-2 nucleocapsid protein [18], IgG/IgM [19] and neutralizing antibody [20]. Colloidal gold nanoparticles (AuNPs) are the most commonly used tags in LFIA for colorimetric detection [21]. However, the traditional AuNPs-based LFIA has limitations in high-sensitivity quantification. Varied novel nanoparticles, such as quantum dots [22], fluorescent microspheres [23], magnetic nanoparticles [24], and up-conversion phosphorescent nanoparticles [25], have been used as reporters in LFIA to break through the limitation. Among them, fluorescence microspheres suffer from narrow Stokes shift, strong background signals, and photobleaching, influencing the accuracy and sensitivity of detection results [26]. After the first introduction of Eu (III) complexes by Weissman [26], the LFIAs with time-resolved fluorescence microspheres (TRFM-based LFIAs) as labels were found to be extremely suitable for rapid on-site detection with high sensitivity. Thus, time-resolved fluorescence microspheres (TRFM), assembled by encapsulating thousands of the lanthanides [27] chelated ions into nano-polystyrene microspheres or silica nanoparticles, have gained popularity among the labels in LFIAs [28]. With the development of a portable time-resolved fluorescence reader, TRFM-based LFIAs have been successfully established and applied in rapid and quantitative detection of antibiotics [29], pathogenic bacteria [30], various food contaminants [31], and disease markers [32].

“Time-resolved” refers to the detection and quantitative analysis of the signal strength of the object to be tested through wavelength resolution and time delay detection techniques [33]. As declared by the previous reports, TRFMs employed as the signal label in LFIAs exhibit good performances, which mostly embody two aspects. On the one hand, TRFMs have excellent fluorescent properties including high quantum yield, extremely wide Stokes shift [34] (200–300 nm), long fluorescence life [35], and narrow and sharp emission spectrum [26], which are benefit to reducing the interference of background fluorescence signals, achieving more sensitive and specific performances. On the other hand, TRFMs modified with abundance carboxylic acid groups have good dispersibility and biocompatibility, which means protein or antibodies can be covalently conjugated onto TRFMs’ surfaces, enhancing the stability of detection probes. Therefore, the TRFM-based LFIAs exhibit good sensitivity and reliability. However, a TRFM-based LFIA for detection of CA199 is rarely reported [2].

In this study, a sandwich format LFIA based on TRFM has been successfully established and employed to detect CA199 in human serum, which combines with a portable reader to provide both qualitative and quantitative results. Overall, the TRFM-based LFIA CA199 detection platform has been demonstrated to be highly sensitive, rapid, accurate, and convenient.

## 2. Results and Discussion

### 2.1. Principle of CA 199 Detection in Serum Using TRFM-Based LFIA

**Figure 1:**
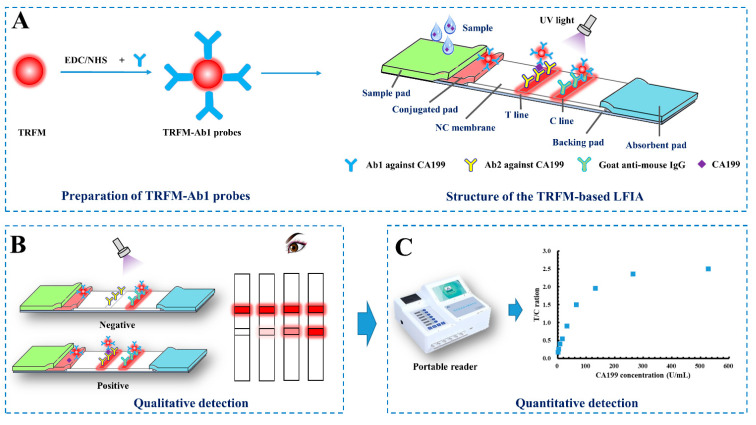
Schematic of the TRFM-based LFIA (**A**) for CA199 rapid qualitative (**B**) and quantitative (**C**) detection.

A sandwich-immunoassay format used in the TRFM-based LFIA is illustrated in Figure 1. Briefly, when CA199 is present in the sample, it would bind with the TRFM-Ab1 probes, and the immune-complex is captured by the Ab2 coated on the T line via immunoreaction, thus forming a “TRFM-Ab1-CA199-Ab2” sandwich and resulting in a fluorescent band on the T line. The excess probes migrate on the NC membrane and bind with the goat anti-mouse IgG coated on the C line, forming another fluorescent band. When CA199 is absent in the sample, only a visible fluorescent band appears on the C line. The qualitative result can be observed under an ultraviolet light, a positive result is presented as two fluorescent lines on the T and C lines, and a negative result is indicated by a single fluorescent band on the C line (Figure 1B). As for quantitative detection, the fluorescent intensity of the T (*FI_t_*) and C lines (*FI_c_*) are obtained and recorded by the portable reader, the ratio between *FI_t_* and *FI_c_* (*FI_t/c_*) is calculated, which can effectively offset the effects of the inherent heterogeneity of test strips and the matrix containing the samples. *FI_t/c_* is proportional to the CA199 level in the sample (Figure 1C).

### 2.2. Optimization of the Parameters

Antibody acts as the key role in the LFIA test strip sensitivity and other performances. As shown in Figure S1A, Ab1 was used to conjugated with TRFM as detection probes, and Ab2 was suitable to be coated on the T line as capture element. To obtain better performances, the amount of Ab1, Ab2, and TRFM-Ab1 probes were optimized. Figure S1B displays that 20 µg of Ab1 was selected to conjugated with TRFMs, the optimal concentration of Ab2 coated on the C line was 0.8 mg/mL (Figure S1C), and the volume of TRFM-Ab1 probes used in a single test strip was 3.0 µL (Figure S1D). The positive samples used in the section was blank serum spiked with 33 U/mL CA199 control material. Visible results of the optimized parameters were provided in Figure S2. The selection of optimized parameters in the study were considered comprehensively including nonspecific reaction, sensitivity, and costing.

The immunoreaction on the TRFM-based LFIA test strip is an instantaneous and dynamic process, which is related to the fluorescence intensity and detection result. Because only a single fluorescence band on the C line appeared when negative sample was detected, the immunoreaction time was investigated by detecting a positive sample (33 U/mL). *FI_t/c_* was recorded by the portable reader every 60 s within 30 min. Figure S3 illustrates that *FI_t/c_* increased with the reaction time but trended to balance and remained basically the same from 15 min to 35 min. It indicated that the detection of CA199 with the developed TRFM-based LFIA can be completed within 15 min.

### 2.3. Assessments of the TRFM-Based LFIA Test Strip Performances

**Figure 2:**
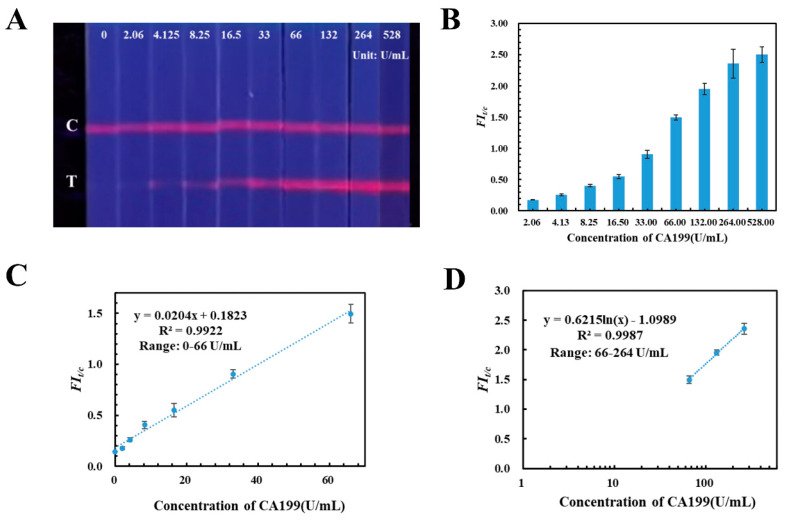
Qualitative and quantitative detection results of the TRFM-based LFIA for CA199. Visible results (**A**) and *FI_t/c_* values (**B**) of TRFM-based LFIA with different concentrations of CA199. The linear ranges for quantitatively detecting CA199 were constructed by plotting the fluorescence ratio versus the concentration 0.00–66.0 U/mL (**C**) and the logarithmic concentration 66.0–264.0 U/mL (**D**), respectively.

Under the optimized parameters, the quantitative detection capability and sensitivity of TRFM-based LFIA test strip was evaluated by fortifying CA199 control material into blank human serum with different concentrations (0.00, 2.06, 4.125, 8.25, 16.5 33.0, 66.0, 132.0, 264.0, 528.0 U/mL), which were detected by the TRFM-based LFIA. Results are shown in Figure 2A: the fluorescent intensity band on the T lines increased with the CA199 control material concentration increasing, and the limit for visible qualitative detection was 4.125 U/mL under an ultraviolet light source with 365 nm wavelength. Then, a calibration curve was plotted by *FI_t/c_* value verse CA199 concentration; Figure 2B displays that the CA199 is positively correlated with *FI_t/c_* value. Furthermore there is a good linear range between them with correlation coefficient at 0.9922 (the corresponding equation is *y* = 0.0204*x* + 0.1823), when the concentration of CA199 was from 0.00 U/mL to 66.0 U/mL (Figure 2C). Additionally, Figure 2D illustrates that the *FI_t/c_* value versus logarithm concentration curve exhibits a certain linear relationship from 66.0 U/mL to 264.0 U/mL, and the correlation coefficient is 0.9987 with equation *y* = 0.6215 *ln*(*x*) − 1.0989. The results indicated that the developed TRFM-based LFIA has the capability to quantitatively detect CA199 in human serum from 0.00 to 264.0 U/mL.

**Figure 3:**
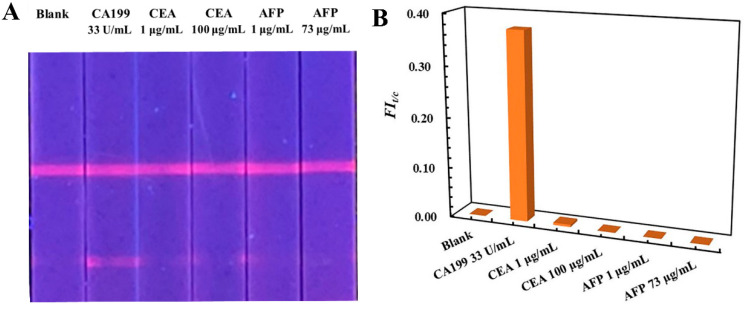
Visible result (**A**) and *FI_t/c_* (**B**) value of the TRFM-based LFIA for specificity evaluation.

The specificity of the developed TRFM-based LFIA was evaluated by analyzing biomarkers’ carcino-embryonic antigen (CEA) and alpha fetoprotein (AFP). Blank serum samples were spiked with 33 U/mL of CA199, 1.0 and 100 µg/mL of CEA, and 1.0 and 73 µg/mL of AFP, respectively. The visible result shown in Figure 3A indicated that a clear fluorescent band appeared on the T line of TRFM-based LFIA test strip when the serum containing 33 U/mL of CA199 was detected, and others exhibited the same as the blank serum sample, and the signal intensity were recorded in Table S1. The *FI_t/c_* value of CA199 is obviously distinguished from that of AFP and CEA (Figure 3B). The results demonstrated that the TRFM-based LFIA for CA199 detection displayed good specificity.

In addition, the precision of the developed TRFM-based LFIA was evaluated based on intra-assay and inter-assay variations. Three concentrations of CA199 (16.5, 33.0, 66.0 U/mL) were tested three times a day for three consecutive days, and relative standard deviation (RSD) of measured *FI_t/c_* value was calculated. As presented in Table 1, the intra-assay and inter-assay RSDs for CA199 detection were less than 3.48% and 10.75%, respectively.

### 2.4. Detection of Human Serum Samples

**Figure 4:**
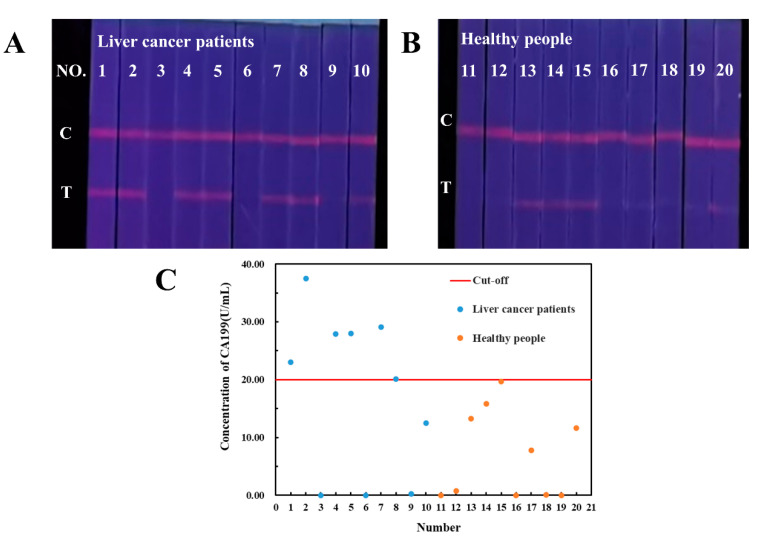
Qualitative results of CA199 in liver cancer patients (**A**) and healthy people (**B**), CA199 levels quantitatively detected by TRFM-based LFIA (**C**).

Human serum samples from 10 liver cancer patients and 10 healthy people obtained from hospital were analyzed by our established TRFM-based LFIA. As shown in Figure 4A,B, CA199 has been detected in human serum of both liver cancer patients and healthy persons. The *FI_t/c_* values were shown in Table S2, and the CA199 concentration in samples was calculated according to the quantitative equations of TRFM-based LFIA. The average level of CA199 in liver cancer patients is higher than that in healthy persons. A CA199 concentration threshold of distinguishing healthy people from liver cancer patients was determined as 20.00 U/mL, and the sensitivity and specificity for liver cancer diagnosis were 60% and 90%, respectively (Figure 4C). Although CA199 is not the specific biomarker for liver cancer, it has certain diagnostic value in the diagnosis and prognosis.

### 2.5. Methods Comparison

**Figure 5:**
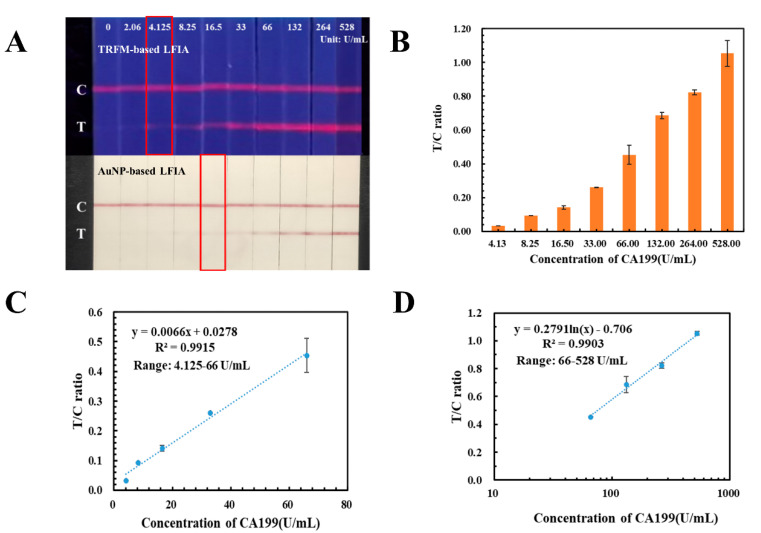
Performances of the AuNP-based LFIA for CA199. (**A**) Comparison between TRFM-based LFIA and AuNP-based LFIA in testing CA199 from 0.00 U/mL to 528.0 U/mL. (**B**) T/C ratio of CA199 detected by the AuNP-based LFIA. (**C**,**D**) Calibration curves for the determination of CA199.

AuNPs were the common signal tracers in LFIA, which had the limitation in high-sensitivity quantification. In this study, an AuNP-based LFIA has also been developed with the same antibody pair against CA199 and applied in detecting the human serum samples with CA199 concentration ranging from 0.00 U/mL to 528.0 U/mL. As displayed in Figure 5A, the detection limit for colorimetric detection of AuNP-based LFIA was 16.5 U/mL, which was four times higher than that of TRFM-based LFIA. Meanwhile, the signal intensities of the T and C lines on the AuNP-based LFIA were recorded by the homemade reader described in our previous study [19], T/C ratio were calculated and displayed in Figure 5B. Calibration and quantification curves were plotted by ratio of signal intensity on T and C lines against the CA199 concentration, the corresponding equations were *y* = 0.0066*x* + 0.0278 (4.125–66.00 U/mL) (Figure 5C) and *y* = 0.2791 *ln*(*x*) – 0.706 (66.0–528.0 U/mL) (Figure 5D), and both of their correlation coefficients were more than 0.99.

Until now, there were only a few LFIAs developed for the rapid detection of CA199 in human serum, and some electrochemical immunological methods also have been reported. The performances of the immunoassays for CA199 detection were comprised and are summarized in Table 2. This indicated that the developed TRFM-based LFIA has the potential for CA199 concentration monitoring in clinical settings, which exhibits simple, fast, convenient, and easy operation.

## 3. Materials and Methods

### 3.1. Chemicals and Reagents

A total of 200 nm of TRFM (excitation: 360 nm, emission: 615 nm) with 1% solid content (*w*/*v*), and 2-(N-morpholino) ethanesulfonic acid (MES) were purchased from Suzhou Vdo Biotech Co., Ltd. (Suzhou, China). Mouse monoclonal antibodies I (Ab1) and II (Ab2) against CA199 were purchased from Nanjing Okay Biotechnology Co., Ltd. (Nanjing, China). Goat anti-mouse IgG was purchased from Beijing Easybio Company (Beijing, China). CA199 control material was purchased from Beijing Ambition Biotechnology Co., Ltd. (Beijing, China). 1-ethyl-3-(3-dimethy laminopropyl) carbodiimide hydrochloride (EDC), N-hydroxysuccinimide (NHS), and Tween-20 were purchased from Aladdin Reagent Co., Ltd. (Shanghai, China). Proclin-300, bovine serum albumin (BSA), D-(+)-Trehalose dihydrate, Sucrose, Polyvinylpyrrolidone (PVP) and Tetronic1307 (S9) were purchased from Sigma-Aldrich (St. Louis, MO, USA). Sample pad (SB08), glass-fiber membrane, PVC pad, and absorbent pad (CH27) were obtained from Kinbio Tech Co., Ltd. (Shanghai, China). Nitrocellulose (NC) membrane (CN95) was purchased from Sartorius (Gottingen, Germany). All solvents and other chemicals were of analytical reagent grade.

### 3.2. Equipments

The XYZ 3D film spraying instrument, CNC cutting machine (CTS300), and microcomputer automatic cutting machine (ZQ2402) were supplied by Kinbio Tech Co., Ltd. (Shanghai, China). Ultrapure water was purified with Milli-Q system from Millipore Corp. (Bedford, MA, USA). The time-resolved fluorescence quantitative analysis reader was purchased from Henan Guanyu Instrument Co., Ltd. (Zhengzhou, China).

### 3.3. Ethics

This study was approved by the Independent Ethics Committee of National GCP Center for Anticancer Drugs (NCC2020C-209) and was conducted from April 2020.

### 3.4. Preparation of the TRFM-Ab1 Detection Probes

Detection probes were prepared by conjugating TRFM with Ab1 via covalent bonds. Briefly, 5 µL TRFM solution (1%, *w*/*v*) was dispersed in 1.0 mL of MES buffer (0.05 M, pH 6.0), and supplemented with 10 µL of EDC and NHS solution (0.5 mg/mL) to activate the carboxyl groups on the TRFM surface. After being fully mixed and shaken for 20 min at room temperature in the dark, it was followed by centrifugation at 9600× *g* for 15 min, and the supernatant was discarded. The precipitate was resuspended in 1.0 mL of phosphate buffer (PB, 0.01 M, pH 7.4). Then, 100 µL of 0.2 mg/mL Ab1 against CA199 dilution was mixed with the activated TRFM and constantly agitated for 2 h at room temperature in the dark. Subsequently, 100 μL of blocking solution (20% BSA) were added to block the unbound sites for 1 h. After centrifugated at 8000× *g* rpm for 15 min, the supernatant was discarded and the precipitate was re-dissolved in 200 μL of dispersant (0.02 M Tris-HCl containing, 0.5% (*w*/*v*) trehalose, 10% (*w*/*v*) sucrose, 0.5% PVP, 0.1% S9, 0.05% Proclin-300, 1% BSA, and 0.1% Tween-20) and stored at 4 °C until use.

### 3.5. Fabrication of the TRFM-Based LFIA Test Strips

The TRFM-based LFIA was assembled by five parts including the sample pad, conjugated pad, NC membrane, absorbent pad, and PVC backing pad. The sample pad was treated with 0.01 M of PBS buffer (containing 0.25% PVP, 0.1% S9, 0.4% Tween-20 and 0.05% ProClin300) for 30 min, and the conjugated pad was immersed in the solution of 0.01 M Tris-HCl buffer containing 4% sucrose, 1% trehalose, and 0.02% ProClin300 for 30 min, both of which were naturally dried for 20 h. Ab2 against CA199 and goat anti-mouse IgG were diluted to 0.8 mg/mL and 0.4 mg/mL with PBS buffer (0.01 M, pH = 7.4), and then sprayed on the NC membrane at a rate of 0.8 μL/cm as test line (T) and control line (C), the distance between T and C lines was 5 mm. Then the NC membrane was dried at 37 °C for 12–16 h and stored in a cool dry place. Subsequently, the treated sample pad, conjugated pad, NC membrane, and absorbent pad were pasted on the PVC backing pad (Figure 1A). Additionally, the fabricated LFIA plate was cut into 3.0 mm-wide test strips, stored at room temperature and kept dry.

### 3.6. Assay Procedure

In total, 10 μL of sample solution were added onto the sample well of the LFIA test strip with 2.0 μL of TRFM-Ab1 probes coated on the conjugated pad, and then 70 μL of 0.01 M PBS buffer (containing 3% NaCl, 1% Tween-20, and 1% BSA) was added to push the probes to migrate on the strip. After 15 min incubation, the qualitative results were observed under an ultraviolet light with a 365 nm filter, the fluorescence intensity of the T and C lines were obtained and recorded by a portable reader (λ_ex_ = 360 nm, λ_em_ = 630 nm).

### 3.7. Optimization of the Parameters

The roles of Ab1 and Ab2 against CA199 in the TRFM-based LFIA have been preliminarily investigated. Different amounts of Ab1 (2.5, 5, 10, 20, 40, 80 µg) were used to conjugate with TRFMs and prepare TRFM-Ab1 detection probes. Ab2 against CA199 was diluted to 0.6 mg/mL, 0.8 mg/mL and 1.0 mg/mL with PBS buffer, and then sprayed on the NC membrane as the test lines. Subsequently, the amount of TRFM-Ab1 detection probes (1.0, 2.0, 3.0 and 4.0 µL) loaded on the conjugated pad was optimized. The optimal parameters were selected by analyzing negative and positive (or spiked) samples.

## 4. Conclusions

In this study, a rapid, sensitive TRFM-based LFIA combined with a portable fluorescence reader has been successfully established for screening CA199 in human serum. The detection limit of the TRFM-based LFIA for visible quantitation under an ultraviolet light was 4.125 U/mL, which was four times lower than that of AuNP-based LFIA. Under the help of the portable reader, it has the capability for specific and quantitative detection within 15 min. According to the results of serum samples from liver cancer patients and healthy persons, 20.00 U/mL of CA199 in human serum was defined as the threshold for distinguishing healthy people from liver cancer patients with an accuracy of about 60%. The proposed TRFM-based LFIA has the advantages of rapid quantification, high sensitivity, and simple operation, pointing to it being potentially useful for point-of-care clinical analysis for biomarkers.

## Figures and Tables

**Table 1:** The precision of the developed TRFM-based LFIA for CA199 detection (*n* = 3).

CA199 Con. (U/mL)	Intra-Assay ^a^			Inter-Assay ^b^		
	Mean Value of *FI_t/c_*	SD	RSD%	Mean Value of *FI_t/c_*	SD	RSD%
16.5	0.632	0.01	1.58	0.620	0.02	3.23
33.0	0.861	0.03	3.48	0.930	0.10	10.75
66.0	1.576	0.01	0.63	1.718	0.17	9.90

**^a^** Tested three times a day. **^b^** Tested three times per day for three consecutive days.

**Table 2:** A summary of immunoassays for detection of CA199.

Method	Nanomaterials	Quantitative	Linear Range	Time (min)	Cut-Off Value	LOD	Reference
Lateral flow	Time-resolved fluorescent mi-crosphere	Yes	12.5–800 U/mL	15	/	6.32 U/mL	Wang et al. [2]
Lateral flow	Gold nanoparticle	Yes	5.0–100 U/mL	20	37 U/mL	5 U/mL	Baryeh et al. [12]
Lateral flow	Magnetized carbon nanotubes	Yes	2.0–200 U/mL	35	37 U/mL	30 U/mL	Huang et al. [36]
Electrochemical sensor	Multiwalled carbon nanotube and magnetite nanoparticle	Yes	0.001–100 ng/mL	30	/	0.163 pg/ mL	Kalyani et al. [37]
UCNP-linked immunosorbent assay	Lanthanide-doped upconversion nanoparticles	No	5.0–2000 U/mL	120	/	/	Zhou et al. [38]
Electrochemiluminescence immunoassay	Quantum dots	Yes	0.005–100 pg/mL	30	/	0.002 pg/mL	Gan et al. [39]
Electrochemical immunosensor	CeO_2_/FeO_x_@mC_500_	Yes	0.1 mU/mL–10 U/mL	/	/	10 µU/mL	Wang et al. [40]
Photothermal immunoassay	Prussian blue nanoparticles	Yes	1.0–100 U/mL	6	/	0.83 U/mL	Han et al. [41]
TRFM-based LFIA	TRFM	Yes	0.0–264.0 U/mL	15	20 U/mL	4.125 U/mL	This work

## Supplementary Materials

The supporting information can be downloaded at: www.mdpi.com/article/10.3390/ijms23179991/s1.
