# Supplementary material for: Lateral Flow Immunoassay Based on Time-Resolved Fluorescence Microspheres for Rapid and Quantitative Screening CA199 in Human Serum
Source: Int J Mol Sci. 2022 Sep 1;23(17):9991. doi: 10.3390/ijms23179991 (PMC9456114; doi:10.3390/ijms23179991)
Supplement: Supplementary file 1 [file ijms-23-09991-s001.zip › ijms-1874179-supplementary.pdf]

Supplementary Information

Lateral flow immunoassay based on time-resolved fluorescence microspheres for rapid and quantitative screening CA199 in human serum

Xueshima Jiao[a,b,#], Tao Peng[b,#], Zhanwei Liang[a,b], Yalin Hu[a,b], Bo Meng[b], Yang Zhao[b], Jie Xie[b], Xiaoyun Gong[b], You Jiang[b], Xiang Fang[b], Xiaoping Yu[a,*], Xinhua Dai[b,*]

[a] *College of Life Sciences, China Jiliang University, Hangzhou, 310018, PR China*

[b] *Center for Advanced Measurement Science, National Institute of Metrology, Beijing 100029, PR China*

# These authors contributed equally to this work.

* Corresponding author. Tel: +86 010 64524208; fax: +86 010 64524962

E-mail address: daixh@nim.ac.cn

yxp@cjlu.edu.cn

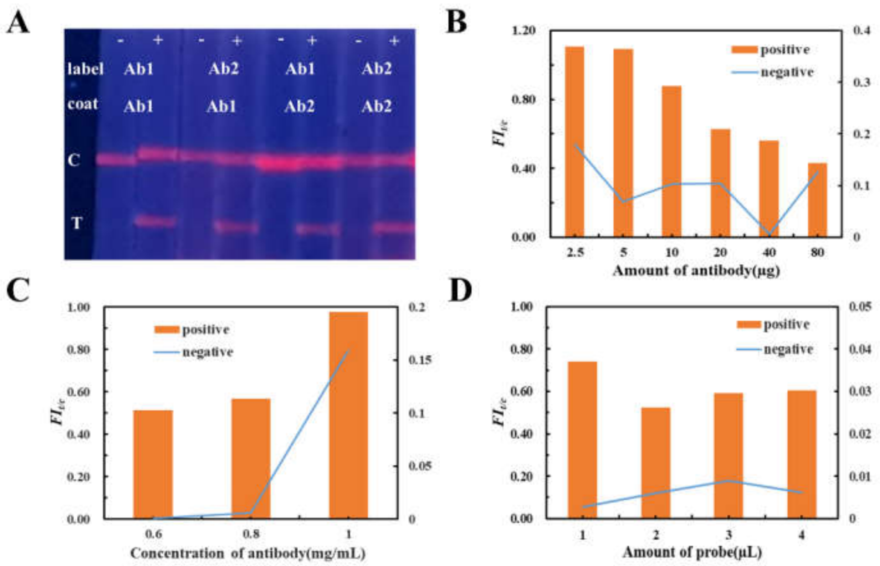

Figure S1. Optimized results of Ab1 and Ab2 roles in the LFIA (A), Ab1 amount used to prepare detection probes (B), Ab2 concentration coated on T line (C), and detection probes volume loaded on conjugated pad (D).

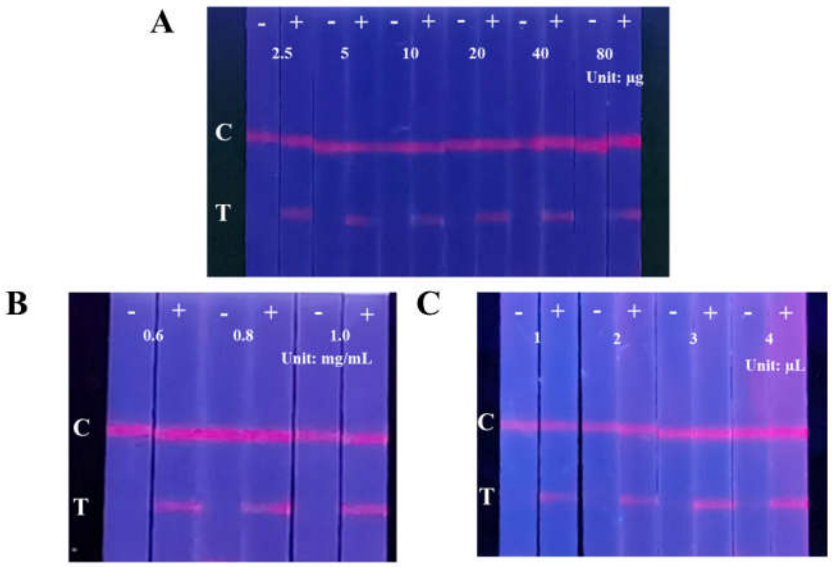

Figure S2. Optimization of (A) the Ab1 in the probe, (B) the Ab2 on the test line, and (C) different probe volumes of 1, 2, 3 and 4 μL.

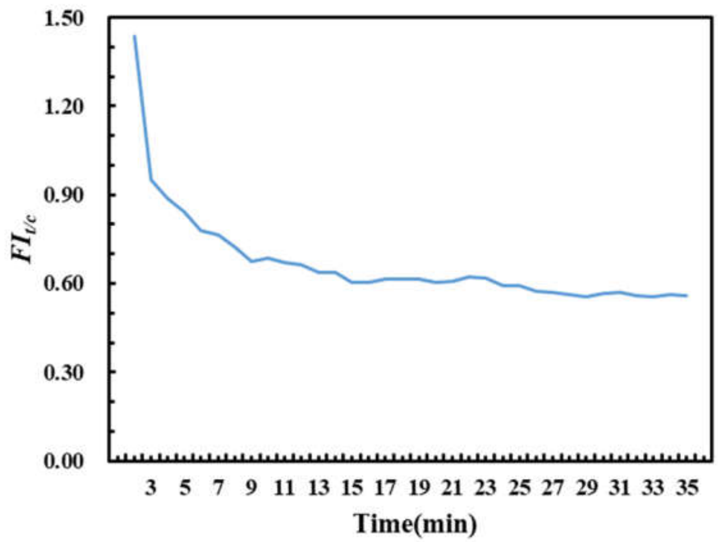

Figure S3. Immunological kinetics analysis of the TRFM-based LFIA for testing 33 U/mL of CA199 in serum

Table S1. The specificity comparison of LFIA strip by different antigens.

Antigens	FT_t	FT_c	$FT_{t/c}$
Blank serum	0.008	16.238	0.000
33 U/mL of CA199	6.975	18.739	0.372
1.0 μg/mL of CEA	0.078	16.982	0.005
100 μg/mL of CEA	0.000	16.907	0.000
1.0 μg/mL of AFP	0.000	17.944	0.000
73 μg/mL of AFP	0.000	17.520	0.000

Table S2. Detection results of CA199 in serum samples of liver cancer patients and healthy people by the TRFM-based LFIA.

Serum samples		FT_t	FT_c	$FT_{t/c}$	CA199 Concentration (U/mL)
Liver cancer patients	1	12.476	19.117	0.653	23.04
	2	15.515	16.383	0.947	37.48
	3	0.225	10.983	0.020	0.00
	4	13.241	17.611	0.752	27.91
	5	13.883	18.421	0.754	27.99
	6	1.148	10.678	0.108	0.00
	7	10.460	13.468	0.777	29.13
	8	9.672	16.313	0.593	20.12
	9	2.007	10.743	0.187	0.21
	10	7.688	17.609	0.437	12.46
Healthy people	11	0.000	13.838	0.000	0.00
	12	3.265	16.51	0.198	0.75
	13	8.954	19.738	0.454	13.29
	14	6.670	13.207	0.505	15.81
	15	9.997	17.141	0.583	19.64
	16	2.952	17.925	0.165	0.00
	17	5.528	16.227	0.341	7.75
	18	2.860	15.532	0.184	0.08
	19	3.853	21.309	0.181	0.00
	20	7.722	18.392	0.420	11.64
